# Preventive effect of reduced glutathione on contrast-induced nephropathy in elderly patients undergoing coronary angiography or intervention: a randomized, controlled trial

**DOI:** 10.1590/1414-431X20154676

**Published:** 2015-07-10

**Authors:** B. Jin, B.W. Wu, J.J. Zhang, X.P. Luo, H.M. Shi

**Affiliations:** Department of Cardiology, Huashan Hospital, Fudan University, Shanghai, China

**Keywords:** Contrast-induced nephropathy, Reduced glutathione, Randomized, controlled trial

## Abstract

Coronary angiography can be a high-risk condition for the incidence of contrast-induced nephropathy (CIN) in elderly patients. Reduced glutathione, under a variety of mechanisms, may prevent CIN in this procedure. We prospectively examined whether hydration with reduced glutathione is superior to hydration alone for prevention of CIN in an elderly Han Chinese population. A total of 505 patients (271 males and 234 females) aged 75 years or older who underwent non-emergency coronary angiography or an intervention were randomly divided into two groups. The treatment group received hydration with reduced glutathione (n=262) and the control group received hydration alone (n=243). Serum creatinine and blood urea nitrogen levels were measured prior to coronary angiography and 48 h after this procedure. The primary endpoint was occurrence of CIN, which was defined as 25% or 44.2 µmol/L above baseline serum creatinine levels 48 h after the procedure. The overall incidence of CIN was 6.49% in the treatment group and 7.41% in the control group, with no significant difference between the groups (P=0.68). In subgroup analysis by percutaneous coronary intervention, no significant differences were found between the two groups. In summary, reduced glutathione added to optimal hydration does not further decrease the risk of CIN in elderly patients undergoing coronary angiography or an intervention.

## Introduction

Contrast-induced nephropathy (CIN) is one of the complications of contrast media, which are used in diagnostic and interventional cardiology procedures. CIN is recognized as an important clinical problem following coronary angiography and percutaneous coronary intervention (PCI) in recent years. Therefore, an increase in the incidence of CIN has resulted in it being the third most common cause of hospital-acquired acute kidney injury ([Bibr B01] [Bibr B02] 13).

Effective prophylactic and therapeutic regimens for decreasing the incidence of CIN are limited. Therefore, additional strategies are urgently required. However, except for intravenous hydration, the optimal strategy for preventing CIN remains uncertain ([Bibr B04] [Bibr B05] 46). Because oxidative stress has been implicated as a contributing factor in the etiology of CIN ([Bibr B07] [Bibr B08] 79), use of a potent antioxidant as a nephroprotective agent is logical. Reduced glutathione, under a variety of mechanisms, may prevent CIN. Limited information is available about the potential preventive benefits of reduced glutathione for CIN in the elderly Han Chinese population. Therefore, we prospectively examined whether hydration with reduced glutathione is superior to hydration alone for prevention of CIN in a randomized, controlled trial.

## Material and Methods

### Study population

This study was carried out at Huashan Hospital, Fudan University between February 2012 and January 2014. Eligibility for the study was defined as patients aged ≥75 years and those who had an estimated glomerular filtration rate ≥60 mL/min/1.73 m^2^ who underwent non-emergency coronary angiography or intervention. Exclusion criteria were acute myocardial infarction, congestive heart failure, and hemodynamic instability during the procedure. A total of 505 patients (271 males and 234 females) aged 75 years or older were eligible. The ethics review board of Huashan Hospital approved the study protocol, and written informed consent was obtained from all participants in the study.

### Study design

This was a non-blinded, randomized, controlled clinical trial among the Han population aged 75 years or older. Consecutive eligible patients were randomly allocated to 2 groups: patients receiving saline plus reduced glutathione (n=262) or saline alone (n=243). Randomization was based upon computer-generated randomization numbers. No placebo was used in this randomized, controlled, clinical trial. The hydration protocol consisted of 1 mL/kg per h of saline for 6 h prior to, during, and 6 h after the procedure. Reduced glutathione (Shanghai Fudan Forward S&T Co., Ltd, China) was administered at the dosage of 2400 mg in saline on the day of the procedure. All of the patients received a non-ionic, iso-osmolar contrast agent (Shanghai Bracco Sine Pharmaceutical Co., Ltd, China) during the procedure. Serum creatinine and blood urea nitrogen levels were measured prior to the procedure and 48 h after the procedure by the same technician in the same laboratory. Paraclinical evaluations were performed in a single hospital laboratory, and laboratory staff were blinded to the study protocol. The primary endpoint of the study was the occurrence of CIN, which was defined as a minimum of 0.5 mg/dL or 25% increase in serum creatinine levels above the baseline 48 h after exposure to contrast media.

### Statistical analysis

Continuous variables with normal distribution are reported as means±SD. Comparisons between the two groups were performed by the Student’s *t*-test. Data analysis was performed with SPSS 12.0. P<0.05 was considered to be significant.

### Results

Demographic data of the patients are summarized in Table 1. The two study groups were generally similar in demographic and baseline characteristics.

**Table:**
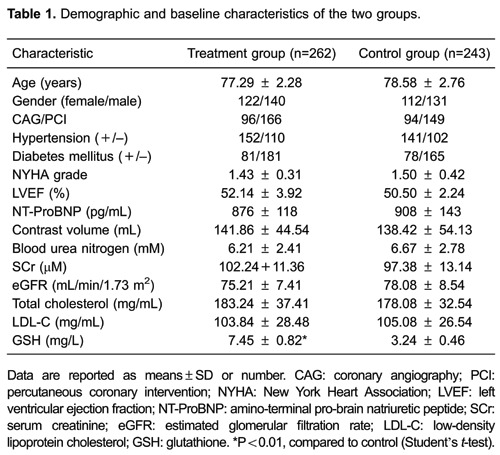


The estimated glomerular filtration rate was not significantly different between the treatment group (72.08±8.31 mL/min/1.73 m^2^) and the hydration group (74.63±9.55 mL/min/1.73 m^2^, Table 2, P=0.64). Similarly, serum creatinine and blood urea nitrogen levels were not significantly different between the two groups after exposure to contrast media.

**Table:**
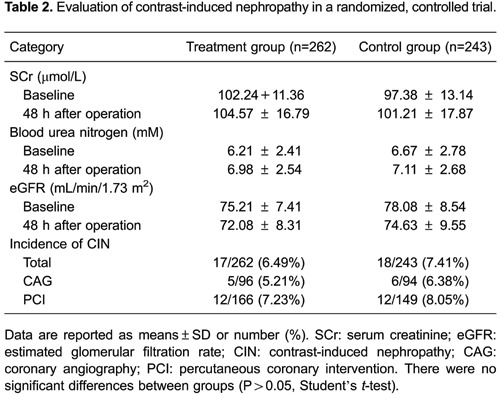


The overall incidence of CIN was 6.93% (35/505) in the Han population aged 75 years or older who underwent coronary angiography or intervention. The incidence of CIN was similar in both groups (6.49% [17/262] in the treatment group versus 7.43% [18/243] in the control group, P=0.68). In subgroup analysis by PCI, no significant differences were found between the two groups (Table 2). No patients required renal replacement therapy during or after the study. There was no mortality in either group during hospitalization.

### Discussion

The main finding of our study was that, although reduced glutathione tended to reduce the occurrence of CIN, this reduction was not significant in elderly patients. Our clinical trial is the first study on the role of reduced glutathione in prevention of CIN in a Han population aged 75 years or older. The patients’ demographic data and baseline risk factors for CIN were similar in the two groups. The overall incidence of CIN is consistent with previous studies in which saline hydration was used as a preventive measure (10,11). Therefore, the lower incidence of CIN with hydration compared with its incidence in medically unprotected conditions is due to the effective hydration protocol.

The pathophysiology of CIN is unclear. Contrast-induced renal dysfunction appears to be due to a reduction in renal blood flow and direct tubular epithelial toxicity (12,13). As a potent antioxidant, reduced glutathione may counteract various pathological mechanisms underlying CIN (14,15). Additionally, glutathione can reduce inflammation, inhibit oxidative stress reactions, and protect the kidney from injury due to complement activation (16,17).

Results of previous studies are controversial regarding the preventive effects of reduced glutathione against CIN (18,19). We found no protective effect of reduced glutathione on serum creatinine levels after contrast material injection. Notably, the present study was conducted on elderly patients with a normal renal function who underwent coronary angiography or intervention.

Even minimal changes in post-procedural serum creatinine levels are associated with increased mortality in patients undergoing coronary angiography (20). Although these changes may be clinically subtle in terms of manifestations, they may signify a great decline in renal function in elderly patients. In the current trial, serum creatinine was studied in both groups. No significant difference in serum creatinine levels was observed between the groups in this study.

Some limitations of our study should be acknowledged. First, the trial was a single-center study, which may reduce its generalizability. Second, the small sample size may have impaired the statistical power of the study in detecting a difference between the groups. Significance might be achieved in larger populations. Third, we did not measure markers of oxidative stress in the present study because of the high cost involved. Finally, short-term administration of hydration with reduced glutathione resulted in a low rate of events in elderly patients with a normal renal function. Further clinical trials in patients with renal impairment are warranted to define the role of hydration with reduced glutathione.

In conclusion, the present study indicated that reduced glutathione added to optimal hydration does not further decrease the risk of CIN in elderly patients undergoing coronary angiography or intervention. However, despite these negative results, a causal relationship may exist in the development of CIN. Well-designed studies with a larger sample size and longer follow-up should be conducted to confirm our results.
